# A quantitative assessment of Arctic shipping in 2010–2014

**DOI:** 10.1038/srep30682

**Published:** 2016-08-01

**Authors:** Victor M. Eguíluz, Juan Fernández-Gracia, Xabier Irigoien, Carlos M. Duarte

**Affiliations:** 1 Instituto de Física Interdisciplinar y Sistemas Complejos IFISC (CSIC-UIB), E07122, Palma de Mallorca, Spain; 2 Harvard T.H. Chan School of Public Health, Department of Epidemiology, 677 Huntington Ave, Boston, MA, 02115, US; 3 King Abdullah University of Science and Technology (KAUST), Red Sea Research Center (RSRC), Thuwal, 23955-6900, Saudi Arabia; 4 University of Tromsø, Faculty of Biosciences, Fisheries and Economics, N-9037 Tromsø, Norway

## Abstract

Rapid loss of sea ice is opening up the Arctic Ocean to shipping, a practice that is forecasted to increase rapidly by 2050 when many models predict that the Arctic Ocean will largely be free of ice toward the end of summer. These forecasts carry considerable uncertainty because Arctic shipping was previously considered too sparse to allow for adequate validation. Here, we provide quantitative evidence that the extent of Arctic shipping in the period 2011–2014 is already significant and that it is concentrated (i) in the Norwegian and Barents Seas, and (ii) predominantly accessed via the Northeast and Northwest Passages. Thick ice along the forecasted direct trans-Arctic route was still present in 2014, preventing transit. Although Arctic shipping remains constrained by the extent of ice coverage, during every September, this coverage is at a minimum, allowing the highest levels of shipping activity. Access to Arctic resources, particularly fisheries, is the most important driver of Arctic shipping thus far.

Opportunities for shipping activities are expanding in the Arctic and aim toward a range of goals, including the assessment and extraction of Arctic marine resources, such as fisheries^1^, minerals, oil and gas^2^, and surveys, tourism^3^ and the investigation of transport along new shipping routes across the Arctic from Asia to Europe and North America^4^,^5^,^6^. Shipping through the Arctic is forecasted to shift global shipping traffic^4^,^5^,^6^, requiring the development of infrastructure and governance arrangements^7^ and the management of risks to marine life and ecosystems^8^. However, to date, analyses have depended on model forecasts, since Arctic shipping and consequently data on Arctic shipping were hitherto too sparse to provide a sound basis for analysis. Recently, shipping traffic has increased considerably in the Arctic Ocean, with tens of thousands of ships operating in the Arctic in 2014.

Here, we examine shipping activity in the Arctic between 2010 and 2014, with a focus on the parcel of ocean extending north from the Arctic Circle (66.4^o^N). We describe the shipping pattern of the Arctic in 2014 in terms of the total number of ships and their distribution to identify areas where shipping concentrates with respect to seasonal patterns. We then compare Arctic shipping in 2014 with that in previous years on the basis of monthly assessments of the spatial distribution of ship density in the Arctic between 2010 and 2014 derived from Automatic Identification System (AIS) data. The AIS data includes the number of unique vessels present each month in cells that cover 1/4 degree of latitude and longitude; vessels are classified as fishing, cargo, tanker, passenger, or “other”. The category “other vessels” includes research vessels, vessels conducting surveys and logistic services for industry, and any other vessel not covered by the preceding explicit categories. Military vessels are not required to report their position through AIS. Our aim is to assess the seasonal and geographical patterns of Arctic shipping, the relationship of these patterns with sea ice extent, and the dominant trajectories of different types of vessels.

A total of 11,066 ships were detected the Arctic in 2014, the majority of which was ships in the “other” category (e.g., supply, research, and survey vessels), followed by fishing (1,960), cargo (1,892), tanker (524), and passenger (308) vessels, where a large fraction is concentrated in the North Atlantic region and only a tiny fraction transited completely through the Arctic. In 2014, Arctic shipping comprised 9.3% of the world’s shipping traffic, a disproportionate 12.4% share of fishing vessels, a 5.9% share of cargo vessels, a 4.2% share of tanker vessels, and a 5.5% share of passenger vessels. The concentration of shipping activity in the Norwegian and Barents Seas (Fig. 1), where an average of over 2,000 vessels were operating per month, made for a highly skewed shipping distribution in the Arctic Ocean (Supplementary Figure 1). The average number of ships per month in a grid cell (*N*_*v*_) depended only mildly on the type of ship, on the geographical and on the temporal aggregation scale (Supplementary Figure 1), which conformed to a power law distribution, *P*(*N*_*v*_) ~ *N*_*v*_^−α^, with an exponent of α = 2.34 (±0.01 SE) for the Arctic. A less steep exponent value compared with that for global shipping distribution, α = 2.15 (±0.01 SE), indicates a less skewed distribution of shipping in the Arctic compared with the global ocean. The spatial distribution of shipping reflects the presence of hot spots of intense shipping, mostly concentrated along the Norwegian and Siberian shelves (Fig. 1), with low-density shipping prevailing elsewhere.

The number of vessels cruising the Arctic declined sharply from the maximum in the Norwegian and Barents Seas, followed by the Siberian and Canadian coasts, to reach a minimum along the Bering-Chukchi Seas (Fig. 1). Fishing activity was predominantly localized in the Barents Sea, and passenger ships operated largely along the Norwegian and Greenland Seas, with Iceland and the Svalbard Islands as ports of call (Figs 1 and 2). In contrast, cargo and tanker ships cruised across the Arctic (Figs 1 and 2). The “other” vessels showed erratic trajectories, entering the area occupied by sea ice, as would be expected from research vessels and industry survey ships (Figs 1 and 2) conducting exploratory activities. As may be expected, high-density areas of transit by shipping correspond with the main Arctic shipping routes: the eastern route along the Northern Sea Route (through the Siberian Sea) from the Bering Sea to the Northeast Passage into the Atlantic Ocean (the dominant route) and the western route, which attracts much less traffic on the Northwest Passage along the Canadian shore (Fig. 1C). The Northwest Passage branches into two routes: a higher-traffic route south of Victoria Island and a lower-traffic route north of Victoria Island. Whereas vessels crossed the permanent ice zone via the North Pole, this transpolar route does not directly link the Bering Sea with the Fram Strait, as predicted by models^4^,^5^,^6^, but rather enters (or exits) the Arctic along the Siberian coast between the Bering Strait and the Lena River estuary to then sail across the North Pole toward the Fram Strait. Along the way, some Arctic harbors attract a disproportionate share of shipping, including harbors far inland along the great Siberian rivers, the small (pop. 1,400) town of Kugluktuk along the Northwest Passage, and the northern tip of Severny Island (Russia).

The monthly assessments of transit along the two main routes, the Northeast and Northwest Passages, show that shipping was concentrated in July-October (Fig. 3). Along the Northeast Passage, the monthly maximum number of cargo and tank vessels increased from 30 in 2011–2013 to 36 in 2014, a 20% increase (Fig. 3A–C and Supplementary Figure 2). A similar calculation reveals that the Northwest Passage was only sporadically transited by one or two cargo vessels (Fig. 3D and Supplementary Figure 3) in a decreasing rather than increasing manner from 2011–2013 to 2014. The number of ships from the “other” category passing along the same longitudes of the two main routes accounted for 50% and 80% of the traffic along the Northeast and Northwest Passages, respectively. Therefore, as of 2014, the Northwest Passage was not regularly used as a shipping route because the sea ice remains too thick for safe shipping^9^ (Fig. 3D and Supplementary Figure 3). On the other hand, the Northeast Passage was continuously used by cargo and tank vessels, with an important increase in shipping in 2014 (maximum of 31 ships in Sept 2012, 32 in Sept 2013, and 49 in Sept 2014). An exponential regression of the reported annual number of transits indicates an average annual increase of 94% (Supplementary Figure 4; a regression of the shipping area to the ice extent and time supports the result of an increase of Artic shipping, see Supplementary Table 1). Although the amount of transit reported is similar to other sources^10^,^11^,^12^, the Northern Sea Route (NSR) Information Office^11^ reported a decrease in the number of vessels transiting the NSR (from 71 transits in 2013 to 53 transits in 2014) in contrast to our data, which indicate a definite increase. Our analysis assumes that ships remain in the same cell as they move eastwards or westwards; that is, a vessel does not move enough in latitude to occupy several cells in the same longitudinal range. The small fluctuation in the number of ships with longitude indicates that this is a reasonable assumption (Fig. 1), although we may risk overestimating the real number of transits. Finally, the traffic from the “other” category accounts for a large fraction of Arctic shipping that cannot be assigned to either of the main routes.

Shipping in the Arctic was strongly seasonal in 2014, with most traffic and occupancy concentrated between July and October (Fig. 4). These seasonal dynamics are driven by seasonal fluctuations in ice coverage^10^,^13^,^14^, whose extent reaches a minimum in September corresponding with a peak in Arctic shipping activity (Fig. 4). The fraction of ice-free open water used for shipping operations was highest in October, when more than 80% of the area free of continuous ice supported shipping, compared with only about 57% in January (Supplementary Figure 5). The seasonal patterns and distribution of Arctic shipping differed greatly among vessel types (Fig. 4). Passenger ships were largely restricted to the summer; fishing, cargo, and tanker vessels moved along the perimeter of the Arctic, rarely passing above 82^o^N; while vessels belonging to the other category ventured into areas occupied by sea ice, approaching the North Pole (Supplementary Figure 6). Shipping declined dramatically north of 75^o^N during all seasons (Supplementary Figure 5), reflecting the coastal nature of the main shipping routes along the coasts of Russia and Canada (Fig. 1c).

In 2014, Arctic shipping was found to occupy between 57 and 80% of ice-free waters. The increased shipping in response to decreased ice coverage in the Arctic observed during 2014 is a reasonable basis for predicting that shipping in the Arctic will continue to increase as the ice coverage decreases^4^,^5^,^6^,^15^,^16^. Although models showing accessibility for transoceanic shipping^4^,^5^,^6^ could support the idea of transoceanic transport as the central factor for shipping in the Arctic, our 2014 results show that access to and exploration of Arctic resources are the overwhelmingly dominant factors determining the extent of Arctic shipping. The availability of quantifiable data on Arctic shipping, and in general global shipping, will allow us to calibrate existing models combining ice extent and real shipping.

## Methods

### Data

AIS systems reporting position through a satellite system are mandatory in all passenger ships irrespective of size, all vessels >300 MT (gross) transiting international routes, and any cargo ship >500 MT transiting within national waters. We do not report on military vessels because they do not require AIS systems. The data analyzed were provided by Exactearth (http://www.exactearth.com/). Values represent the monthly number of unique vessels within grid cells that cover 1/4 degree of latitude and longitude; vessels were classified into five categories: cargo, tank, passenger, fishing, and other.

### Grid cell area

Because the area encompassed by grid cells changes considerably with latitude (i.e., the cell size increases as the latitude decreases towards zero degrees) we report vessel density as the number of vessels per unit area. The area of a grid cell was calculated using a radius of R = 6371 km (Earth’s radius). Thus, for a grid cell whose center is located at latitude *φ*, the area of the grid cell is given by*A*_*φ*_ = 2*ΔθR*^2^cos(*φ*)sin(*Δφ*/2), where *Δθ* and *Δφ* correspond to the width in longitude and latitude of the grid cell in radians. In our case, the grid cells cover 1/4 degree of longitude and latitude and thus


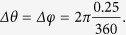


### Exponents of the distribution of the number of vessels

The exponents were determined using a maximum likelihood fit to a power law^17^.

### Ice extent

Information on ice extent, obtained from the National Snow and Ice Data Center^18^ (http://nsidc.org/data/G02135), was available as monthly averages since 1979. The data is frequently updated with the latest sea-ice data and can be downloaded in the form of shape-files containing either the line that defines the ice border or as the polygons that define the regions covered by sea ice. The files are free for download through the ftp ftp://sidads.colorado.edu/DATASETS/NOAA/G02135/shapefiles/. We assessed whether each cell of the grid from the vessel data was covered by ice in each month.

### Transits along the Northwest and Northeast Passages

To visualize the most used routes in the Arctic, we identified the two grid cells with the highest average monthly traffic for each longitude and linked them longitudinally. While evaluating the number of vessels passing along these routes, we found that the longitudinal shipping slightly fluctuates around an average value and thus we quantify the transit as the aggregated number of vessels for a given longitude averaged between 90^o^ and 180^o^ E for the Northeast Passage and between 200^o^ and 245^o^ E for the Northwest Passage.

## Additional Information

**How to cite this article**: Eguíluz, V. M. *et al*. A quantitative assessment of Arctic shipping in 2010–2014. *Sci. Rep*. **6**, 30682; doi: 10.1038/srep30682 (2016).

## Supplementary Material

Supplementary Information

## Figures and Tables

**Figure 1:**
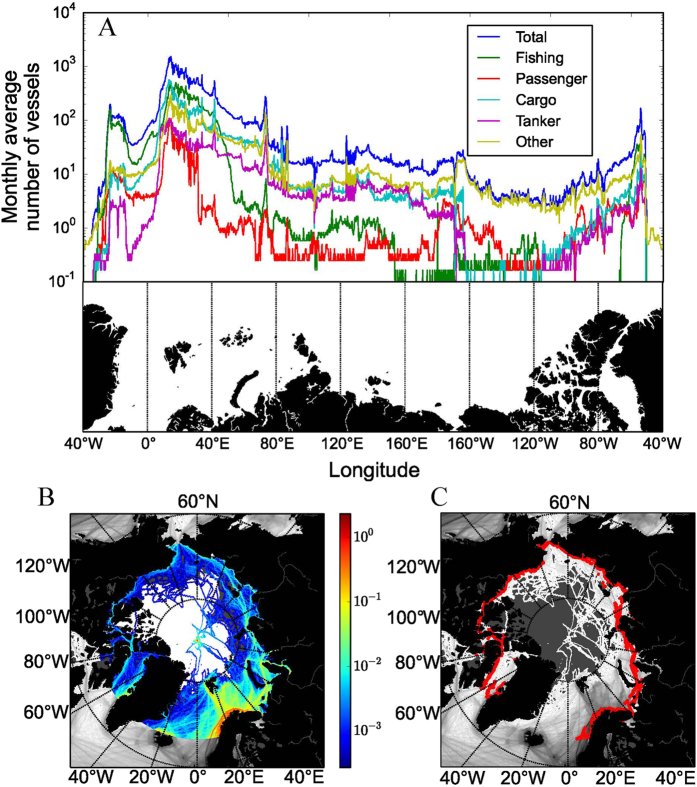
A global view of shipping in the Arctic. Monthly average (**A**) number and (**B**) density of ships (units: number of vessels per square kilometer) present in the Arctic Ocean at different longitudes and (**C**) the main shipping routes in the Arctic in 2014. The number of ships in (**A**) represents the number of ships at any latitude >66.4^o^N for 1/4 degree longitude sectors around the Arctic Circle, and the colors denote the type of ship. (**B**) Heat map displaying the average number of ships per square kilometer and month during 2014 in a 1/4 × 1/4 degree cell grid. The increase in density toward the North Pole is due to the decrease of the area covered by each grid cell with increasing latitude while observing the same number of ships. Ice cover in September 1014 is represented in white. (**C**) Main navigation routes in the Arctic (in red) obtained by identifying the two most densely grid cells per month on average for each 1/4 degree longitude bands (see Methods), The monthly average density from (**B**) is also shown in grey scale (ice is not shown). Figure generated with python 3.x (https://www.python.org/) and PyShp (https://pypi.python.org/pypi/pyshp).

**Figure 2:**
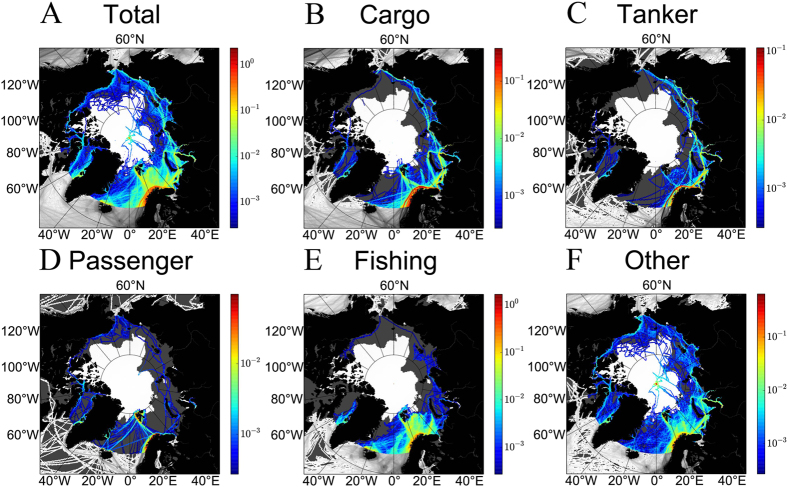
Average monthly densities of ships per ship type. The color scale is logarithmic; ice cover represents that during September, when sea ice coverage is smallest. Figure generated with python 3.x (https://www.python.org/) and PyShp (https://pypi.python.org/pypi/pyshp).

**Figure 3:**
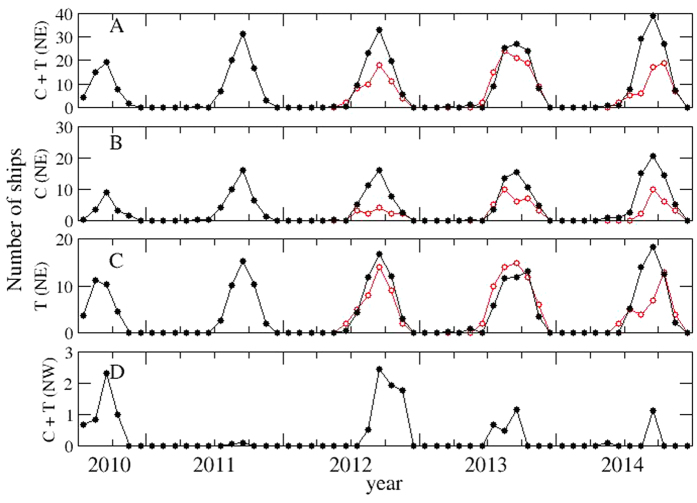
Transiting the Arctic. Monthly average number of vessels along the two main Arctic routes: (**A**–**C**) the Northeast (NE) and D) Northwest (NW) Passages. For the Northeast Passage, the average number of ships per month shows a seasonal pattern that is highest between June-November with a maximum in September ((**A)**: cargo and tank, (**B**) cargo, and (**C**) tank). Shipping by cargo and tanker vessels increased by 20% in 2014 compared to the 2012 maximum. For completeness, we also display the values obtained from the report of the Northern Sea Route^14^ (empty red circles). (**D**) The Northwest Passage only shows one or two cargo or tank vessels transiting each year between 2012 and 2014. The number of ships from the “other” category account for around 50% of the traffic in the longitudes corresponding to the Northeast Passage and around 80% of those corresponding to the Northwest Passage (Supplementary Figures 2 and 3).

**Figure 4:**
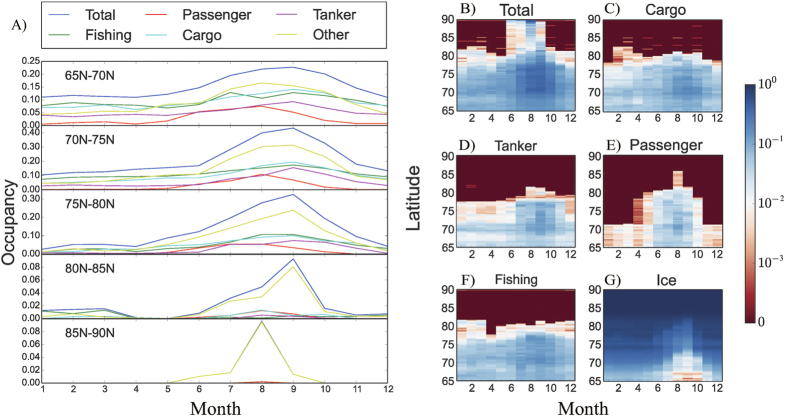
Shipping per latitude, month, and vessel type. (**A**) Percentage of area covered by ships as a function of month for latitude in the range 65–70^o^N, 70–75^o^N, 75–80^o^N, 80–85^o^N, and 85–90^o^N. (**B–F**) Percentage (in logarithmic scale) of area covered by ships per month, latitude, and vessel type: total (**B**), cargo (**C**), tank (**D**), passenger (**E**), and fishing (**F**). (**G**) The percentage of ice coverage with month.
