# Supplementary material for: A quantitative assessment of Arctic shipping in 2010–2014
Source: Sci Rep. 2016 Aug 1;6:30682. doi: 10.1038/srep30682 (PMC4967913; doi:10.1038/srep30682)
Supplement: Supplementary Information [file srep30682-s1.pdf]

Supplementary Information

A quantitative assessment of Arctic shipping in 2010-2014

Victor M. Eguíluz, Juan Fernández-Gracia, Xabier Irigoien, and Carlos M. Duarte

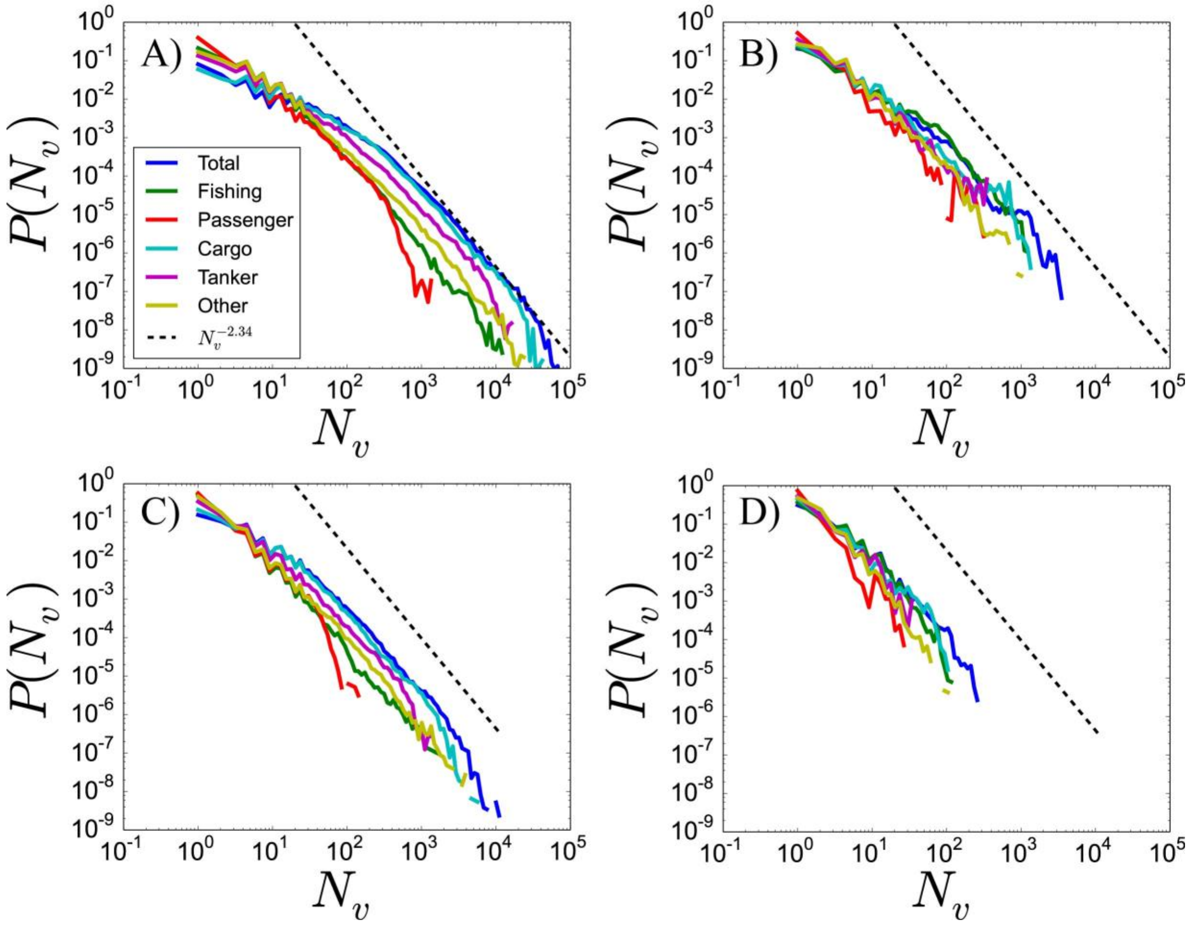

Supplementary Figure 1. **Distribution of the number of unique vessels per grid cell**. (First row) Annual aggregate (A) worldwide and (B) in the Arctic; (second row) for September (C) worldwide and (D) in the Arctic. The dashed black line is a guide to the eye showing a power-law decay of the probability to find N_v vessels in a grid cell: $P(N_v)\sim N_v^{-\alpha}$ with α=2.34 for (a) and (c) and α=2.15 for (b) and (d).

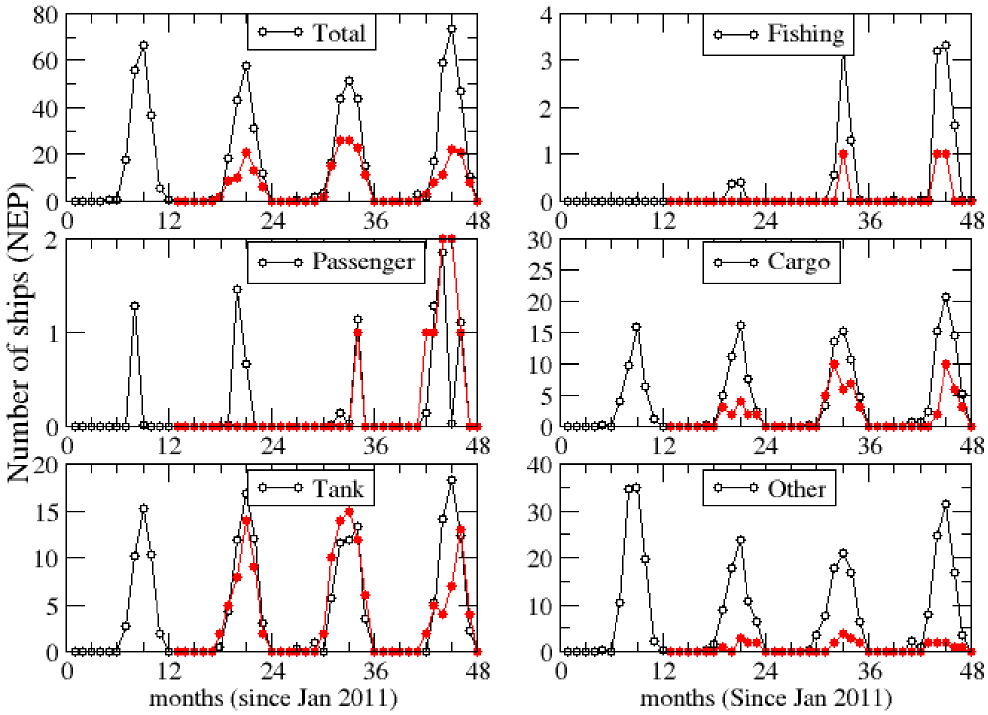

Supplementary Figure 2. **Shipping along the Northeast Passage (NEP).** Average number of different types of vessels ships along the Northeast Passage (empty symbols) and the number of transits along the Northern Sea Route (filled symbol) between 2011 and 2014. The average number of ships is calculated by first aggregating the number of vessels per grid cell for latitudes larger than 66.5 °N and then averaging for longitudes in the range 90-180 °E. The number of transits along the Northern Sea Route were obtained from NSR Information Office reports[1].

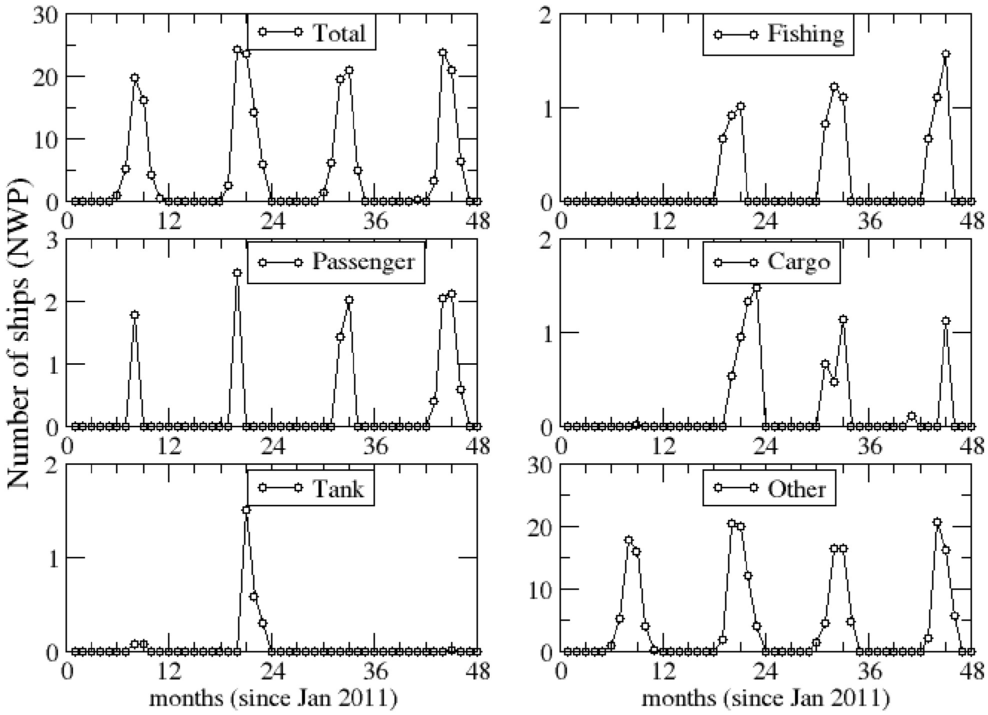

Supplementary Figure 3. **Shipping along the Northwest Passage (NWP).** Average number of different types of vessels along the Northeast Passage between 2011 and 2014. The average number of ships is calculated by first aggregating the number of vessels per grid cell for latitudes larger than 66.5 °N and then averaging for longitudes in the range 200-245 °E.

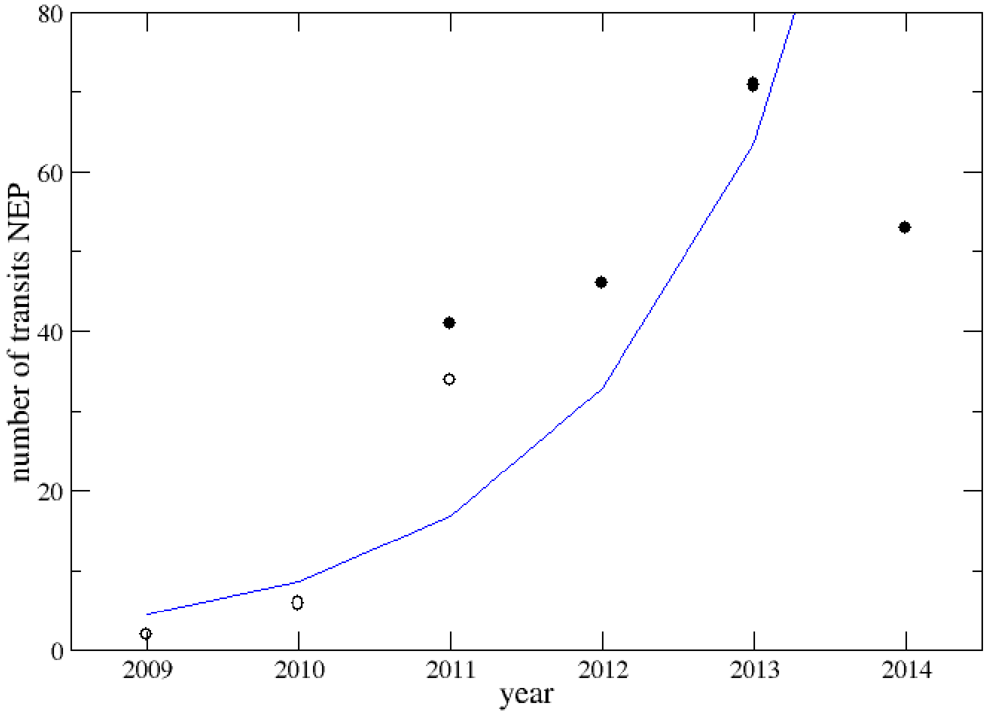

	2009	2010	2011	2012	2013	2014
Ref. 1			41	46	71	53
Ref. 2	2	6	34			
Ref. 3		4	34	46		

Supplementary Figure 4. **Annual transits in the Northeast Passage (NEP) between 2009 and 2014.** Data correspond to values reported by Ref. 1 between 2011 and 2014 (filled symbols), and by Ref. 2 between 2009 and 2011 (empty symbols). Note: the values in Ref. 2 may refer to vessels rather than to transits. An exponential fitting of the form e^{at} (continuous line) gives a characteristic parameter a=.66 ±18 (SD), which corresponds to a relative annual increase of 94%. Alternatively a linear regression gives an average annual increase of 13±3 vessels per year (a correlation coefficient of 0.89 for the linear regression and an exponential regression of 0.85).

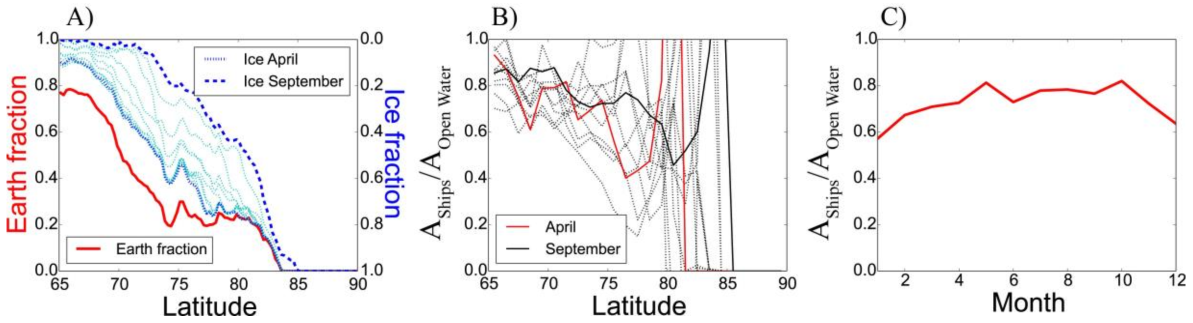

Supplementary Figure 5. **Percentage of Arctic occupancy and ice extent.** (A) Earth fraction (red, scale on left axis) and ice occupancy (blue, scale on right axis) as a function of latitude. Ice[4] is represented for April (dotted) and September (dashed) of 2014 by highlighting in darker blue, as they are the months with most and least ice, respectively. The lighter dotted blue curves correspond to the remaining months. The space between the red and blue curves shows the fraction of open water. (B) The ratio of grid cells occupied by ship (A_{Ships}) and by sea ($A_{Open\ Water}$) as a function of latitude. Values larger than 1 indicate navigation across the ice. April (solid red line) and September (solid black line) of 2014 are highlighted, as they are the months with more and less sea ice, respectively. The dotted curves show the rest of the months. (C) The ratio of area covered by ships (A_{Ships}) and open water ($A_{Open\ Water}$) in the entire Arctic sea as a function of month.

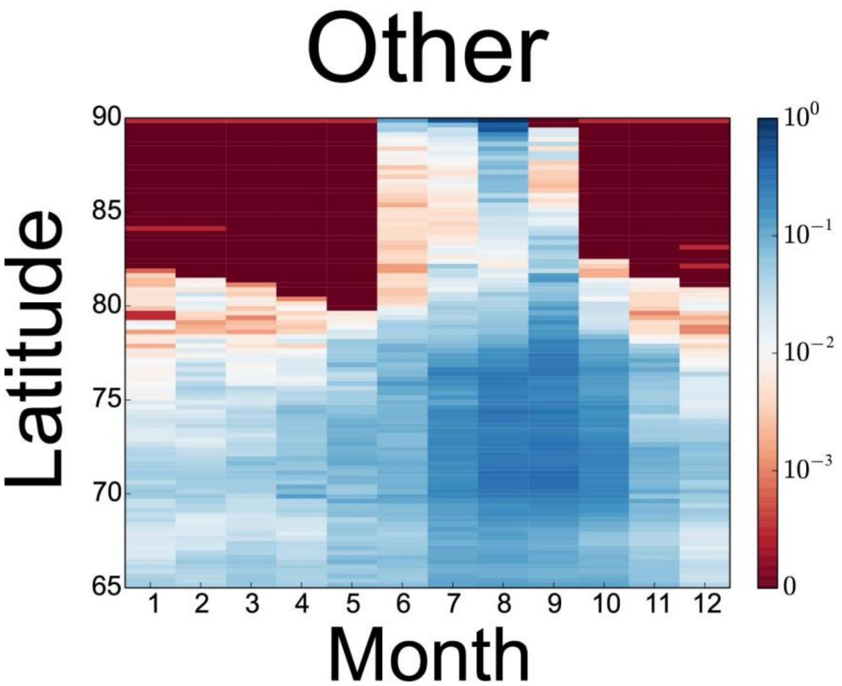

Supplementary Figure 6. **Arctic occupancy per month with latitude.** Percentage of occupied grid cells as a function of the month with latitude for the vessels under the category of other.

Type of vessels	β ± CI	γ ± CI ($10^3 km^2$/year)	R^2
Total	**-0.70 ± 0.04**	**126 ± 58**	0.96
Fishing	**-0.164 ± 0.024**	**95 ± 39**	0.80
Passenger	**-0.15 ± 0.04**	40 ± 61	0.57
Cargo	**-0.26 ± 0.02**	**120 ± 33**	0.93
Tanker	**-0.22 ± 0.02**	**37 ± 29**	0.93
Other	**-0.60 ± 0.05**	40 ± 73	0.93

Supplementary Table 1. **Regression of area occupied by vessels with time and area covered by ice.** We analyzed the monthly data from June 2010 to December 2014 on ice extent and shipping by vessel type. We perform a regression of the area occupied by each type of vessel, A_{vtype}, as a function of time and ice area, $A_{vtype}=\alpha+\beta A_{ice}+\gamma t$, where α is the intercept and is an offset to the statistical model needed to fit the data; β is the effect of the ice covered area and is interpreted as the extra units of area covered by the specific type of vessels when an extra area unit is covered by ice, given that time is kept fixed (this coefficient is expected to be negative); and γ is the effect of time and is interpreted as the extra area units covered by the specific type of vessels when a time unit is added, keeping the ice covered area constant. If the area covered by shipping is only driven by the ice-covered area, i.e., by the available free-ice area, γ can't be statistically different from 0. On the contrary, if γ is statistically different from 0 there is a temporal increase (for $\gamma>0$) [decrease, for $\gamma<0$] in the shipping area, independent on ice-coverage. Furthermore we will characterize the goodness of the fit to the data by R^2, which measures the percentage of variation in the data explained by the statistical model. The fitting values show that the shipping area increases with decreasing ice

coverage (β=-0.70 ± 0.04) and increases with time at a rate γ=126 ± 58 $10^3 km^2$/year. The coefficients obtained depending on the type of vessel are shown in the table.

References

1. http://www.arctic-lio.com/nsr_transits (Accessed Jan 4, 2016).

2. Østreng, W. et al. Shipping in Arctic Waters, Springer, Heidelberg (2013).

3. Stephenson, S.R., Brigham, L. W. & Smith, L. C. Marine accessibility along Russia's Northern Sea Route. *Polar Geography* **37**, 111-133 (2014).

4. Fetterer, F., Knowles, K., Meier, W. & Savoie, M. Sea Ice Index, Version 1. Boulder, Colorado USA. NSIDC: National Snow and Ice Data Center. http://dx.doi.org/10.7265/N5QJ7F7W (2002, updated daily)
